# Multimodality Treatment Options and Outcomes of Laryngeal Carcinosarcoma: A Clinical Analysis of a Rare Tumor from a Single Hospital

**DOI:** 10.1155/2019/1754675

**Published:** 2019-06-16

**Authors:** Yang Zhang, Zhigang Huang, Neil Gross, Jugao Fang, Xiaohong Chen, Xuejun Chen, Lizhen Hou, Pingdong Li, Guojun Li, Qi Zhong

**Affiliations:** ^1^Department of Otolaryngology, Head and Neck Surgery, Beijing Tongren Hospital, Capital Medical University, Beijing 100730, China; ^2^Key Laboratory of Otolaryngology Head and Neck Surgery (Capital Medical University), Ministry of Education, Beijing 100730, China; ^3^Key Laboratory of Molecular Pathology of Head and Neck, Beijing 100730, China; ^4^Department of Head and Neck Surgery, The University of Texas MD Anderson Cancer Center, Houston, TX, USA; ^5^Department of Epidemiology, The University of Texas MD Anderson Cancer Center, Houston, TX, USA

## Abstract

Primary laryngeal carcinosarcoma is a rare type of malignancies, and the standard therapeutic protocol for its treatment has yet to be established. This retrospective study analyzed the clinical and pathological characteristics, risk factors, treatment options, and prognosis of 13 patients with primary laryngeal carcinosarcomas. This case series included 11 males and 2 females with an age range from 32 to 78 years at diagnosis. The initial clinical symptoms included hoarseness, dyspnea, and foreign body sensation in the throat. The primary tumor sites were at the glottis, supraglottis, and larynx. All these patients were active or passive smokers, and more than half of them were alcohol addictive. The surgical treatment for laryngeal carcinosarcomas included CO2 laser surgery in seven cases, partial laryngectomy and neck dissection in three cases, and total laryngectomy and neck dissection in three cases. Among them, seven cases received postoperative radiotherapy. After follow-up with a mean of 31.15-month, four cases died. Primary laryngeal carcinosarcoma is a rare but more aggressive malignancy. Contralateral lymph node metastasis can occur at the early stage of this disease. A treatment combining surgery and postoperative radiotherapy is strongly recommended.

## 1. Introduction

Carcinosarcoma, also known as sarcomatoid carcinoma or spindle cell carcinoma, is a relatively rare type of malignant tumor. Carcinosarcoma has been reported under a wide variety of pathological names, including carcinosarcoma, spindle cell carcinoma, Lane tumors (squamous cell carcinoma with pseudosarcoma, Lane tumor), pleomorphic carcinoma, metaplastic carcinoma, polypoid squamous cell carcinoma, pseudocarcinosarcoma, and carcinoma with pseudosarcoma. In the clinic, carcinosarcoma is often found in sites of the upper gastrointestinal/respiratory tract, such as head and neck mucosa, the throat, and esophagus entrance. However, carcinosarcoma of the larynx is rare and only represents 2%-3% of throat cancers [1–3]. Because of its rarity, there is currently no generally acceptable treatment guideline for this disease.

In this study, we retrospectively enrolled 13 cases with laryngeal carcinosarcomas to analyze the clinical manifestations, diagnosis, and treatment, as well as treatment outcome of patients. These patients were diagnosed with laryngeal carcinosarcoma within the past ten years in our hospital.

## 2. Materials and Methods

A total of 13 cases were retrospectively recruited and they were diagnosed with laryngeal carcinosarcomas from 2005 to 2014 at the Beijing Tongren Hospital. The clinical information was collected from the medical records. The HE and immunohistologically stained sections were re-reviewed by two pathologists to confirm the diagnosis. Other available data including gender, age, clinical presentation, tumor site, tumor subtype, treatment, treatment outcome, follow-up, and vital status were reviewed and collected from the patients' medical records. Among these cases, information on endoscopy, imaging tests, and pathological examination was available for analysis.

The follow-up of the patients ended in October 2018. Overall survival (OS) was defined as the time from first diagnosis to death from any cause or date of last follow-up. Participants who were alive at the end of the study period or lost to follow-up were considered censored. Medical record review for follow-up status of all patients was performed under direct supervision of attending head and neck surgeon. Primary tumor site, clinical stage, treatment, and vital status were reviewed from medical records as assessed between the initial and final patients contacts recorded. All histological samples were reconfirmed by one senior pathologist. Survival analysis was performed using the Kaplan-Meier method, reported as mean with 95% confidence intervals (CI). SPSS software, version 16.0 (SPSS, Inc., Chicago, IL, USA), was used for the statistical analyses. The Ethics Committee of the Beijing Tongren Hospital approved the protocol of this study.

## 3. Results

### 3.1. Patients' Characteristics

A total of 13 laryngeal carcinosarcomas, including 11 from males and 2 from females, were included in this study. The age range at diagnosis was 32 to 78 years with a median age of 58 years. All males were smokers and have smoked for more than 15 years. All females were passive smokers. Seven cases had alcohol addiction. The initial clinical symptoms of 13 laryngeal carcinosarcomas included hoarseness in 8 cases, dyspnea in 3 cases, and foreign body sensation in the throat in 2 cases. All cases had hoarseness before surgery including 3 cases having symptoms of dyspnea. Other symptoms that appeared before surgery included foreign body sensation in the throat in 4 cases, sore throat in one case, and neck masses in 3 cases. As shown in Table 1, the primary tumor sites were located at the glottis in 11 cases (including 3 cases in the hypopharynx) and supraglottis in 2 cases (including one case in the epiglottis and one case in ventricle of larynx). The TNM staging of 13 laryngeal carcinosarcomas included two cases with T1, five with T2, and six with T3 stage. Eight cases were at N0, one was at N1, and 4 were at N2 stage. No distant metastasis was observed before surgery. Two cases were at clinical phase stage I, 4 were at stage II, 2 were at stage III, and 5 were at stage IV.

### 3.2. Patients' Treatment

The surgery of laryngeal carcinosarcomas included a CO2 laser surgery in seven cases, a partial laryngectomy and neck dissection in three cases, and a total laryngectomy and neck dissection in three cases. Among the seven cases that received CO2 laser surgery, two patients underwent neck dissection with laser surgery, where one case had lateral recurrence after surgery and underwent partial laryngectomy plus neck dissection, and the other case had contralateral recurrence after surgery and underwent a total laryngectomy plus ipsilateral neck dissection (Table 1). Among the three cases who underwent partial laryngectomy plus neck dissection, one case had contralateral lymph node metastasis 10 months after surgery and underwent contralateral neck dissection. Among the three cases that received total laryngectomy plus neck dissection, one case had contralateral lymph node metastasis three months after surgery and underwent contralateral neck dissection. Seven cases received postoperative radiotherapy at a dose of 66-80 Gy (Table 1).

### 3.3. Patients' Follow-Up

**Figure 1:**
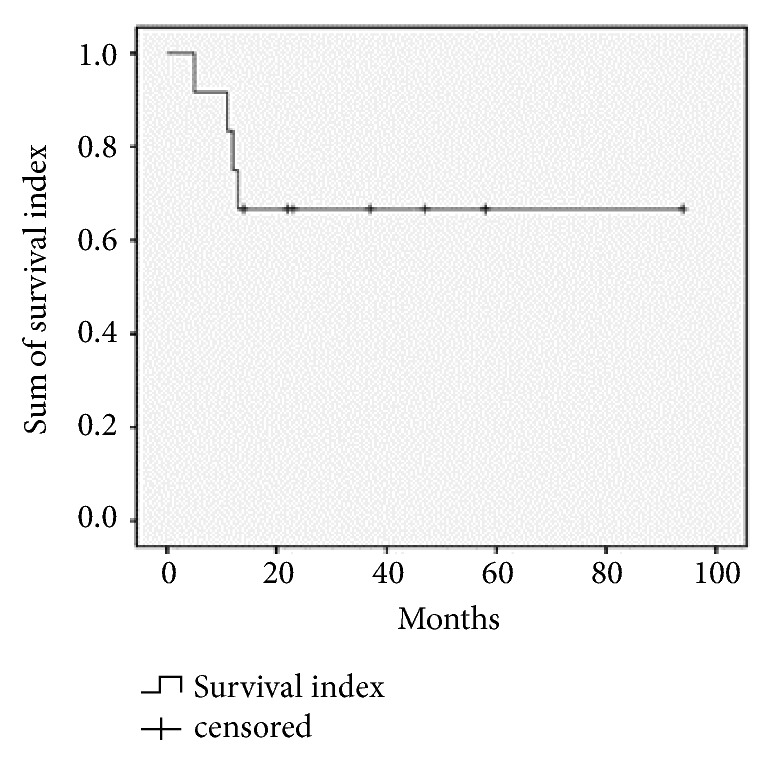
Survival curve of 13 patients with primary laryngeal carcinosarcoma.

The median follow-up time was 22 months (range: 5-94 months) with a mean follow-up time of 31.15 months. Four cases that did not receive radiotherapy died. Among these four cases, case one had a glottic T3N2M0 tumor, underwent total laryngectomy plus right neck dissection, and died from recurrence at 12 months after surgery. Case two had a glottic T3N1M0 tumor, underwent total laryngectomy plus both side neck radiation, and died from recurrence at five months after surgery. Case three had glottic T2N0M0 tumor, underwent minimally invasive laryngoscopy, and died from recurrence at 13 months after surgery. Case four had a glottic T2N0M0 tumor, underwent MLLS plus double neck radiation, and died from recurrence at 12 months after surgery. Nine patients survived at the end of follow-up and had no recurrence. Among the 9 alive patients, seven cases received postoperative radiotherapy, and two cases did not accept postoperative radiotherapy. Of the two cases without radiotherapy, one case had supraglottic T1N0M0 tumor, underwent minimally invasive laryngoscope, and survived with recurrence. The other case had supraglottic T2N0M0 tumor, first underwent minimally invasive laryngoscopy, then underwent a total throat plus ipsilateral neck dissection three months after first surgery due to contralateral recurrence, and survived for more than 12 months. As shown in Figure 1, four patients died between 5 to 13 months after surgery and nine patients survived without recurrence for 11 to 94 months.

## 4. Discussion

Most publications on carcinosarcoma are case series reports [4–6]. While laryngeal carcinosarcoma is even much rarer, there is no standard protocol for the treatment of this rare disease. A previous report demonstrated that the majority (93.0%) of patients with laryngeal carcinosarcoma are male [3]. In our patients with laryngeal carcinosarcoma, the ratio of men to women is 11:2. Previous studies suggested that alcohol addiction, smoking, and radiation exposure are more likely the major risk factors for laryngeal carcinosarcoma, and approximately 87% and 48% of these patients had a history of smoking and drinking, respectively [3, 7]. In the current study all patients were active or passive smokers, with more than 15 years of smoking. Moreover, over half of the patients had alcohol addiction, while all patients had no clear history of radiation exposure. The most common sites of primary laryngeal carcinosarcomas are the vocal cords, followed by the glottis. Almost all patients in this study experienced hoarseness and some patients presented with shortness of breath as the main symptom. The associated symptoms included foreign body sensation in the throat, sore throat, and neck masses. No patients had a T4-stage tumor at diagnosis, but about half of the cases had T3-stage tumors. Furthermore, five patients had lymph node metastasis but no distant metastasis. However, approximately 80% of cases with lymph node metastasis had a N2-stage tumors.

Currently in the literature, surgery is the main treatment for primary laryngeal carcinosarcoma. In this study, the procedures included minimally invasive laryngoscopy, partial laryngectomy, and total laryngectomy. According to our experience, T1 and T2 tumors can be treated with minimally invasive laryngoscopy, but patients should be closely followed up. If postoperative recurrence occurs, surgery and radiotherapy are the recommended options for treatment. The T3- and T4-stage tumors should be treated with partial or total laryngectomy. We found that lymph node metastasis was not very common, but the metastasis for early stage was observed, such as T1-stage glottic tumors. Moreover, there was a tendency to metastasize to the opposite side of the neck. This study suggests that patients with T1-stage glottic tumors, even though they are N0-stage tumors, should undergo prophylactic neck dissection. Generally, the minimally invasive laryngoscopy plus dissection of ipsilateral neck II and III area should be adequate, and the bilateral modified radical neck dissection should be considered if the intraoperative frozen pathological examination suggested lymph node metastasis.

Currently, whether radiotherapy is an optional treatment for primary laryngeal carcinosarcoma still remains controversial. In the study by Zhang et al., postoperative radiotherapy is not recommended for treatment as mesenchymal tumor cells are not sensitive to radiation [7]. In contrast, another study by Ballo et al. suggested that radiation therapy can be used as a single treatment and can effectively treat carcinosarcoma [8]. In the present study, two of the four patients that died had clinical stage II and IV and did not receive postoperative radiotherapy. Thus, we recommend postoperative radiotherapy for patients with a clinical stage higher than stage II after a full excision of regional lymph nodes.

A previous study suggested that the prognosis of patients with laryngeal carcinosarcoma is worse than patients with normal laryngeal squamous cell carcinoma [7]. Our study also showed a poor prognosis in laryngeal carcinosarcoma patients. A previous study proposed that the negative factors associated with poor prognosis of laryngeal carcinosarcoma patients include tumor T-stage, tumor location, vocal cord movement, and history of head and neck radiation therapy, as well as whether necrosis appeared [3]. In contrast, the patient's sex, age, tumor growth pattern, presence or absence of fibrous connective tissue proliferation, and cell degradation had no effects on prognosis [3]. In this study, due to the small number of cases and the majority of tumors being at supraglottis, the above described characteristics were not observed, while the T-stage and vocal cord movement may have effect on prognosis.

In summary, primary laryngeal carcinosarcoma is a very rare malignancy with a tendency to occur with contralateral lymph nodes metastasis at early stage of the disease. A treatment combining surgery and postoperative radiotherapy is recommended.

## Figures and Tables

**Table 1:** Clinicopathological characteristics and treatment options of laryngeal carcinosarcoma.

Sex	Age (years)	Tumor sites	Stage	Treatment	Follow-up
M	65	glottis	T1N0M0	laser + Rad 66 Gy	DFS, 47 months
M	59	glottis	T2N0M0	laser +Rad 80 Gy	DFS, 22 months
M	45	supraglottis	T2N0M0	First: laser Second: PL+WND+Rad 66 Gy	DFS, 94 months
F	62	glottis	T3N0M0	PL+RND+Rad 66 Gy	DFS, 58 months
M	56	glottis	T3N0M0	TL+WND+Rad 66 Gy	DFS, 11 months
M	35	glottis	T3N2M0	First: PL+LND+Rad 70 Gy Second: RND	DFS, 37 months
F	58	supraglottis	T3N2M0	TL+WND+Rad 70 Gy	DFS, 23 months
M	60	glottis	T1N0M0	laser	DFS, 58month
M	69	glottis	T2N0M0	First: laser Second: TL+RND	DFS, 14 months
M	78	glottis	T2N0M0	laser	Died, 13 months
M	32	glottis	T2N0M0	laser +WND	Died, 12 months
M	43	glottis	T3N1M0	TL+WND	Died, 5 months
M	58	glottis	T3N2M0	TL+RND	Died, 11 months

Laser: CO2 laser surgery; Rad: radiotherapy; PL: partial laryngectomy; TL: total laryngectomy; LND: left neck dissection; RND: right neck dissection; WND: whole neck dissection; DFS: disease-free survival.

## Data Availability

The data in this manuscript was collected from patients' medical records.

## Abbreviations

Rad: Radiotherapy
PL: Partial laryngectomy
TL: Total laryngectomy
LND: Left neck dissection
RND: Right neck dissection
WND: Whole neck dissection
DFS: Disease-free survival.
